# Transnational Dynamics Amid Poor Regulations: Taiwan’s Asbestos Ban Actions and Experiences

**DOI:** 10.3390/ijerph14101240

**Published:** 2017-10-17

**Authors:** Harry Yi-Jui Wu, Ro-Ting Lin, Jung-Der Wang, Yawen Cheng

**Affiliations:** 1 Medical Ethics and Humanities Unit, Li Ka Shing Faculty of Medicine, The University of Hong Kong, Hong Kong, China; hyjw@hku.hk; 2 Department of Occupational Safety and Health, College of Public Health, China Medical University, Taichung 40402, Taiwan; 3 Department of Public Health College of Medicine, National Cheng Kung University, Tainan 70101, Taiwan; jdwang121@gmail.com; 4 Institute of Health Policy and Management, College of Public Health, National Taiwan University, Taipei 10055, Taiwan; ycheng@ntu.edu.tw

**Keywords:** asbestos, asbestos-related diseases, health policy, Taiwan

## Abstract

This article describes the history of the asbestos use regulation process in Taiwan and the associated factors leading to its total ban in 2018. Despite the long history of asbestos mining and manufacturing since the Japanese colonial period, attempts to understand the impact of asbestos on the health of the population and to control its use did not emerge until the early 1980s. We attempted to investigate the driving forces and obstructions involved in asbestos regulations by reviewing available public sources and scientific journal articles and conducting interviews with key propagators of the asbestos regulation and ban. Correlation between asbestos exposure and asbestos-related diseases has already been established; however, authorities have been unable to effectively regulate the extensive application of asbestos in various light industries that support economic growth since the 1960s. More stringent regulations on asbestos use in industries and an eventual ban were caused indirectly by appeals made by visionary scholars and healthcare professionals but also due to the subsidence of asbestos-related industries. With the elucidation of factors that affect asbestos regulation and ban, a thorough long-term healthcare plan for the neglected victims of asbestos-related diseases and upstream measures for policy change must be developed.

## 1. Introduction

The implementation of the national policy leading to the total asbestos ban in Taiwan on 1 January 2018 marks a historic moment for the country [1]. Such a ban reveals a health policy-making process resulting from the transnational dynamics among public health scientists. Asbestos has long been widely used in the construction and manufacturing industries [2]. Asbestos use in Taiwan varied as the types of industry changed during the past century. Various ways of asbestos utilization and levels of asbestos exposure are related to its effects on human health. Meticulous efforts have been conducted in Taiwan to establish the relationship between asbestos exposure and asbestos-related diseases (ARDs); however, regulations and the final total ban on asbestos depend largely on growing environmental awareness, transnational networking among advocates, and the decline of asbestos-related industries. Asbestos remains an environmental hazard despite the forthcoming stringent regulations on its industrial use.

**Figure 1:**
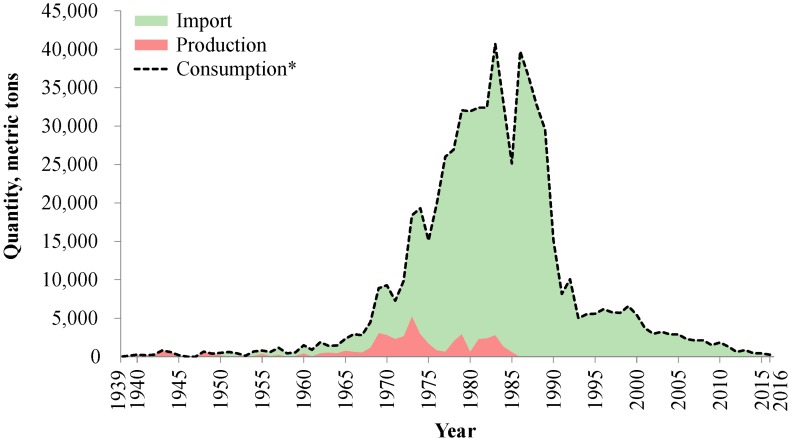
Trends of unmanufactured asbestos import, production, and consumption in Taiwan, 1930 to 2016 [5,15,16,17,18,19]. * Consumption = Production + Import.

The industrial application of asbestos in Taiwan can be divided into three stages: before 1945 (under Japanese rule), 1945–1983 (the development of the fundamental, light, and heavy industry), and 1984–2017 (high-tech industry development). Asbestos deposits were first discovered on the east coast of Taiwan in 1917 and were mined on a large scale to support Japanese industrial development plans from 1937 to 1944 [3,4]. The asbestos mine produced 105–820 metric tons asbestos per year in this period (Figure 1) [5]. Since 1945, with the end of Japanese rule and the Second World War, Taiwan entered a period of economic development. Taiwan imported asbestos extensively from South Africa and Canada [6]. Most unmanufactured asbestos was consumed in four sectors: asbestos cement, abrasion resistant products, insulation, and textiles [7]. Meanwhile, Taiwan dominated the worldwide ship demolition industry to extract raw materials from ships to support domestic industrial development [8]; however, data on asbestos-containing materials (ACMs) from these demolished vessels are lacking.

Asbestos consumption peaked in 1983 and gradually decreased following the growing awareness about asbestos hazards, the establishment of asbestos regulations, the industrial transition to the manufacture of high-tech products, and decreasing demand for asbestos products from Taiwan’s trade partners [9,10]. Consumption suddenly peaked again in 1986 for house reconstruction after the devastating typhoon Wayne [11,12]. Most asbestos was consumed in the mid-1980s, but no regulation was established to prohibit asbestos consumption until 1989.

Establishing asbestos regulations was an important factor that accounted for the decrease in asbestos consumption after the 1980s, whereas the efforts of public health scholars to stimulate the awareness of asbestos workers about asbestos’ hazards were another critical factor that contributed to the drastic decline in asbestos use. During this period, officially registered factories that operated with asbestos were subjected to asbestos regulations, which enabled public health scholars to enter these factories and conduct a national survey on asbestos exposure among workers who handled asbestos textiles, cement, insulation, and brake-lining materials [13,14]. In addition, the presence of investigators equipped with the highest level of personal protective equipment (PPE) to conduct air samplings surprised many workers and employers of the inspected asbestos factories during the time when many workers and employers lacked awareness of the health risks from asbestos exposure.

## 2. Asbestos-Related Regulations in Taiwan

Internationally, Selikoff et al. raised concern about elevated mesothelioma risks among insulation workers, mostly due to the occupational exposure to asbestos, in the 1960s [20]. Since then, knowledge of the health impact of exposure to asbestos has emerged and grown among asbestos-consuming countries. Taiwan’s regulations pertaining to asbestos control were finally announced by the Ministry of Labor (MOL, formerly named the Council of Labor Affairs) in 1981 and the Environmental Protection Administration (EPA) in 1989, respectively. Taiwan spent 37 years regulating asbestos, from first establishing a permissible exposure limit (PEL) in 1981 to the total ban policy in 2018.

In 1981, the MOL established the first asbestos-related regulation, i.e., the PEL for asbestos in the workplace was set at 5 fiber/cc of air as a time-weighted average [21]. In 1982, the government initiated a project wherein it collaborated with occupational health scholars to collect air samples from workplaces with asbestos operations [13,14,22]. The report revealed stunning results that the average exposure level was as high as 4.85 ± 3.11 fiber/cc, and more than 75% of the collected samples exceeded the PEL [22]. Since then, concerns about the impact of exposure to asbestos on health have emerged among workers and residents living nearby those factories [23]. Driven by academia, the government initiated another project to assist several factories that manufactured asbestos cement sheet to improve their working environment from 1988 to 1990 [22].

To understand the extent of manufacture, distribution, use, storage, and disposal of asbestos in Taiwan, the EPA passed the amendment of Toxic Chemical Substances Control Act (TCSCA) in 1989 that require facilities that engage in handling (i.e., activities involving manufacture, import, export, sale, transport, use, storage, or disposal) ACMs ≥ 15% *w*/*w* to comply with applicable rules under TCSCA [24,25,26,27,28,29,30,31,32]. Table 1 summarizes the history of asbestos authorization and prohibition by Taiwan EPA.

In the 1990s, several case reports on ARDs, i.e., pleural plaque and asbestosis, were linked to historical asbestos exposure in the workplace [33,34]. Further governmental actions for asbestos restriction were undertaken during this period. The EPA included asbestos in the Authorization List; therefore, asbestos has since become subject to authorization and cannot be handled unless authorized for a specific use [24,25,26,27,28,29,30,31,32]. In 1997, EPA announced to ban the use of crocidolite and amosite, except for research, testing, or education purposes [24,25,26,27,28,29,30,31,32]. However, the ban was only a symbolical amendment, as Figure 1 shows that no obvious change was observed in asbestos consumption before and after 1997 [18,19], probably because most consumed asbestos was chrysotile rather than crocidolite and amosite.

In the 2000s, international restrictions on the marketing and use of asbestos became important drivers of propelling regulatory changes, including the European Union’s (EU) directive 1999/77/EC and Japan’s Kubota Shock in 2005 [35,36]. The EU’s directive prohibited ACMs from the market and new applications starting 1 January 2005 [35]. The dispute settlement of the World Trade Organization in favor of the French ban on chrysotile was a landmark for asbestos-importing countries to implement a total ban [37]. Japan’s Kubota Shock was the discovery of a heavy outbreak of ARDs among former workers in an asbestos-cement pipe plant, as well as the residents living nearby the manufacturing facility in the summer of 2005 [36]. With its historical and geographic proximity to Japan, Taiwan has been one of the important trade partners of Japan and also followed many Japanese regulations. After the Kubota Shock, Taiwan EPA, which had been pressured by local academics and environmental health activists, issued a notification that manufacturing of asbestos plate, pipe, cement, and asbestos fiber cement board would be banned on 1 January 2008. However, unsatisfied with the passive responses of the government on regulating other ACMs, local public health academics urged the government to adopt a total asbestos ban in several international conferences since 2009. Ever since, growing media reports and public awareness propelled the Taiwan EPA to speed up regulations (from 10 authorized operations to one authorized operation, see Table 1). Finally, Taiwan decided to phase out asbestos starting 1 January 2018, although companies with permits obtained before 1 January 2018 can still handle asbestos until the expiration date [1,38].

## 3. Driving Forces of Regulatory Change and Total Ban on Asbestos

Factors that facilitated the insidious changes in regulations and the final total ban on asbestos in Taiwan are multifold. In the 1980s, growing awareness of environmental causes led to the establishment of the Bureau of Environmental Protection (which became EPA in 1987) at the state level in 1982; and in the following year, 5 fiber/cc PEL of asbestos was mandated by the MOL. The establishment of PEL provided a guideline for academic research. Towards the end of the 1980s, the MOL invited public health scholars to participate in the plan to investigate and improve the working environment for asbestos workers. After the mid-1990s, with the establishment of the professional body regarding environmental and occupational medicine, scholars started to systemically investigate the association between asbestos exposure and ARDs [34]. However, with the scarcity of available epidemiological evidence and case reports, occupational health scholars had to rely on transnational networks to enhance their advocacy for promoting the asbestos ban. Ultimately, it was the decline in asbestos-related industries that accelerated the effectiveness of the total asbestos ban policy in Taiwan.

### 3.1. Academic Research

Scholarly efforts in Taiwan to prove the correlation between asbestos exposure and illnesses were indispensable. The correlation between asbestos and ARDs had been established in the United States in the 1960s; however, neither nationwide exposure assessments nor PEL regulations in Taiwan occurred during that period. After the establishment of PEL, several reports that highlighted workplace asbestos exposures that exceeded PEL were conducted in the 1980s [13,14]. Occupational health scholars visited factories in the 1980s and they deliberately wore full PPE to educate factory owners and plaintiffs regarding the potential harm from the asbestos. In the 1990s, with the institutionalization of environmental and occupational medicine, sporadic case reports of ARDs related to the job nature of asbestos exposure started emerging [33,34]. However, epidemiological evidence for asbestos workers remained limited before the early 2000s because of the poor accessibility of labor insurance and health database for scholars. A historical investigation suggested that only the Labor Insurance possessed the statistical information on occupational diseases, such as silicosis and asbestosis, in Taiwan and these data were not open for academic research [39]. In the 2010s, increasing epidemiological evidence showed high risk of respiratory cancer among workers associated with occupational asbestos exposure [8,40,41,42,43]. ARDs, including asbestosis, pleural plaques, and mesothelioma, remained under-recognized in Taiwan [38]. The lack of evidence might have arisen from the nature of ARDs, which involve a necessary lag period of up to 3–4 decades. It nevertheless did not stop public health scholars in Taiwan from taking the lead to call for a total asbestos ban.

### 3.2. International Trends of Total Ban

International trends regarding asbestos regulations have indirectly influenced similar works in Taiwan. In 1977, the International Agency for Research on Cancer (IARC) concluded sufficient evidence to list asbestos as a carcinogen [44,45]. The timing became a watershed moment that affected the trade of asbestos-related products between Taiwan and other countries. However, light industries that were required for the economic development of the state remained one of the most important hindrances to asbestos regulation. In the 1980s, although the United States started to curtail imports of unmanufactured asbestos from Taiwan, products that contained asbestos were still highly demanded in the trade market [9]. In 2005, the renowned Kubota event in Japan became a milestone for the Japanese government to recognize the responsibility of the state in ARDs. It further facilitated the creation of related compensation laws in Japan. An international proposal for the collaboration on eliminating the use of asbestos and preventing ARDs was proclaimed by the International Labour Organization (ILO) and World Health Organization (WHO) in the 13th Session of the Joint ILO/WHO Committee on Occupational Health in 2003, which later was approved as the ILO Congress Resolution concerning asbestos in 2006 [46,47]. In 2006, the World Health Organization (WHO) made an official call to eliminate ARDs [48]. The call was made amid the global tension related to the industries [49]. Although Taiwan was excluded from the list of WHO member states, efforts were taken to respond to the global trend. However, government authorities occasionally partnered with the asbestos industry in promoting asbestos use. For example, Canada used to be one of the biggest sources of asbestos for industries in Taiwan in 2007; therefore, Taiwan EPA hosted the “Chrysotile International Scientific Workshop” with Canadian Trade Office in Taipei to promote safe use of asbestos, rather than prohibiting asbestos [50]. Such effort was still deemed by public health scholars as a treacherous action conspired by authorities in the vested interest to maintain asbestos use. Canada used to be the main source of raw asbestos for Taiwan to manufacture asbestos-containing products, such as asbestos pipes and fabric and friction materials [18]; after 2012, China replaced Canada and became the largest supplying country of raw asbestos for Taiwan [18]. On the contrary, Taiwan exported asbestos-containing products mainly to China and ASEAN countries [18].

### 3.3. Incomplete Regulations and Transnational Advocacy

Constrained by the shortage of epidemiological evidence, authorities in Taiwan have not been effective in implementing the regulations on asbestos use in industries. Unlike France and Japan, no legal actions have been undertaken to urge employers or governmental sectors to establish useful compensation mechanisms for occupational diseases with long latency periods [38]. Such reluctance points to the factor of time. In Taiwan, a large scale asbestos exposure among workers of the construction and shipbreaking industries occurred during the 1970s and 1980s. Their onset of symptoms mostly did not occur until the 2000s and beyond. Despite the institutionalization of EPA and occupational medicine in the 1980s, subjects of epidemiological studies remained sporadic. In addition, although from 1958, workers’ compensation benefits have been available for victims of occupational accidents through the labor insurance scheme [51], oral history evidence shows that victims were mostly either reluctant or non-eligible to apply for the compensation benefits [38]. The direct causes of not initiating a workers’ compensation claim were the lack of knowledge about causation of disease and the unawareness of workers’ rights to compensation for occupational disease [38]. The indirect causes were perceived difficulties in claiming process, fear for potential hostility from employers, and regulatory barriers [38]. The regulatory barriers of current labor insurance system, for example, didn’t provide the compensation benefits to those non-eligible workers, including self-employed workers, small-sized enterprise employees, and retirees [38]. This reveals the flaws of current insurance design, which has insufficient coverage rate and does not account for the nature of ARDs and difficulties of claiming compensation for occupational diseases [38].

Experience in Taiwan to a certain degree echoes the finding of an interdisciplinary team that investigated the history of disease recognition and compensation for silicosis [52]. Taking South Africa as an example, they found that under the condition of semi-colonization, the downside of incomplete regulation on occupational hazards could be remedied by transnational dynamics of advocacy works [52]. When industries did not act in full power regarding employee safety and health, the recognition of occupational diseases and corresponding exposures to workplace hazards became relatively easier [52]. Thus, the work of disease recognition and compensation could bypass the constraints of science, which usually required strong causal relationships between occupational exposure and diseases [52]. From the year the PEL of asbestos exposure was regulated, asbestos usage was selectively allowed in several industrial categories in Taiwan, covering nearly the major areas of asbestos-correlated commerce. From the late 2000s, scholars of public health and occupational medicine as well as local NGOs have been attempting to improve the visibility of agenda related to the adverse health effects of asbestos in Taiwan. On the 15th of May 2009, participants from five countries signed the Taipei Declaration document in Taiwan at the International Conference on Industrial Risks, Labor and Public Health in Taipei. On the 25th of April 2010, another petition was called for at the EPICOH-Medichem conference in Taiwan. On 22 November 2011, the second round of Taipei Declaration was issued at the 10th Asia Pacific NGO’s Environmental Conference in Taiwan, joined by experts from Taiwan, France, and Japan, to call the governments in the Asian region taking actions for a total asbestos ban. In December 2015, a local NGO, i.e., the Taiwan Occupational Safety and Health Link, together with public health scholars (including the authors, J.D. Wang and Y. Cheng) organized a campaign workshop. The workshop asked the government to regulate ACMs and asbestos-containing waste, to establish a surveillance system of ARDs, to reform the labor compensation systems, and to increase public awareness about asbestos and its health risks. The growing social media coverage and public awareness about asbestos problems drew legislators’ attention. On 23 June 2016, a full-day legislative session was undertaken in the Legislative Yuan, where several legislators questioned administration departments on asbestos problems. The newly appointed minister of Taiwan EPA (Dr. Ying-Yuan Lee), who holds a strong educational background in public health and is quite aware of the urgency and significance of this issue of banning asbestos finally announced the plan of total ban on asbestos in 2018 [1].

## 4. Discussion

Many industrialized countries had experienced difficulties in implementing a total ban due to the unique contribution of asbestos to economic activity. Increasing public concerns and accumulated scientific evidence have indicated the link between the mineral and serious health effects; therefore, many countries have reoriented national policies toward global trends in prohibiting all types and forms of asbestos to eliminate ARDs [53]. Taiwan will be the 62nd country to implement a national policy on the total asbestos ban [53]. The ban will mark the start of ARDs elimination in Taiwan.

Exposure to asbestos will persist in the coming years and even decades. Asbestos still exists in roof tiles and boards in old construction structures. Urban renewal has been among the priorities of the government in recent years; thus, increasing asbestos-containing waste is foreseeable [54]. However, Taiwan’s regulations on managing residual asbestos or existing ACMs remain incomplete. The government has not established a nationwide system for monitoring, labeling, and managing residual or existing asbestos, particularly in the construction industry. This poses the risk of inflicting ARDs among workers engaging in the construction, demolition, and waste handling, as well as the residents living nearby areas with those activities. In addition, previous Australian cases of importing building products that contained asbestos from countries that have not prohibited asbestos have attracted increasing attentions and efforts towards reliable checking of ACM issues [55].

International evidence has indicated a clear association between the amount of historical asbestos and the number of deaths from ARDs [56]. Global models have predicted the asbestos-to-mesothelioma ratio ranging 170–331 metric tons per case and asbestos-related lung cancer-to-mesothelioma ratio of 6.77 [57,58,59]. It should be noted that these numbers varied with cumulative asbestos consumption, latency periods, study periods, and the number of reporting years [57,58,59]. For example, the asbestos-to-mesothelioma ratio could drop to 109 metric tons per case by dividing the latest estimate of global mesothelioma, i.e., 38,400 annual deaths, by the average of global asbestos consumption, i.e., 4.18 million metric tons during 1970–1990 [17,60]. However, regulations related to the exposure and health examination of workers do not cover several high-risk groups, such as house demolition and boiler maintenance workers, because most of these workers are contractors or self-employed and are therefore not subject to the regulations. Even for formal employees, the regulation requires employers to keep health-related data for 30 or more years [61], but most factories are either no longer existing or not keeping historical records on exposure and health examination. In addition, only 33 factories that used asbestos have been officially registered and subjected to local regulation during the 1980s [13,14], thereby indicating that a few “underground factories” were not included in the official factory inspections for safety and health across Taiwan. A nationwide surveillance system that combines geographical information system, asbestos consumption data, and working history should be established to capture the exposure extent. Moreover, such system must also cover current construction and/or maintenance workers that deal with residual ACMs and nearby residents.

The long latency period of ARDs makes the identification of occupational diseases highly difficult, especially under the current system that requires only on-the-job workers to participate in the occupational health examination (OHE) program in Taiwan. The government of Taiwan should expand the scope of the OHE program to include workers with histories of occupational exposure to asbestos to establish a periodic surveillance system of mesothelioma and other ARDs. Furthermore, the compensation system of workers should require major reforms to adequately recognize and compensate victims of occupational diseases.

In this paper, we showed the obstructive and facilitating factors regarding asbestos regulations and the eventual total ban in Taiwan. Recognition of ARDs remains difficult based on current legal and insurance system and the vision that ARDs will, by nature, become an environmental disease from an occupational one; therefore, the government should expand the coverage of compensation to workers that experienced job transfers, resignations, and retirement. Recognition and compensation of ARDs should be acknowledged at a life-long period. In addition to the provision of long-term care for patients, upstream opportunities regarding policy changes, including establishing a nationwide system to survey asbestos exposure and ARDs, should not be missed. Measures should be taken to enhance the awareness among citizens and stimulate corporate responsibilities regarding the prevention of asbestos emissions inside and outside the workplace.

## 5. Key Messages

- The impact of asbestos on health was identified in the 1980s in Taiwan, but the state spent 37 years to the implementation the total ban policy starting 1 January 2018.
- Our study highlights four important driving forces involved in asbestos regulations in Taiwan, including the institutionalization of environmental and occupational medicine, appeals made by public health scholars, transnational advocacy works, and the transformation of industry.
- The long-term health effects of residual asbestos and long-term care for affected patients should be recognized and included in the asbestos policy reform.

## 6. Conclusions

This study outlines the social, economic, and scientific forces that hinder and facilitate the total asbestos ban in Taiwan. The relationship between asbestos and ARDs has been established, but the insidious manifestation and indefinite symptoms of ARDs, personal attribution of diseases to hazard exposure, and extensive need for asbestos in light industries have hampered the progress of asbestos regulation. The total asbestos ban in Taiwan is contingent on multifold factors, including the institutionalization of environmental and occupational medicine, appeals made by public health scholars, transnational advocacy works by health activists, and subsiding of related industries during economic transformation of Taiwan. Banning asbestos is the very first step to prevent asbestos exposure. As a result of protracted consumption and late ban, actions should be taken regarding the long-term care for affected patients and the upstream measures for policy reform.

## Figures and Tables

**Table 1:** Regulations covering asbestos authorization and prohibition in Taiwan ^1^.

Date	Requirements
1989.05.01	Listed asbestos-containing materials (ACMs) ≥ 15% *w*/*w* as one of chemicals subject to Toxic Chemical Substances Control Act Prohibited new installation of asbestos pipes
1989.11.07	Exempted existing products containing asbestos < 15% *w*/*w*
1991.02.27	Prohibited new installations of drinking water pipes containing asbestos.
1996.10.17	Announced only authorized operations could handle asbestos, including #1, #2, #3(a), #3(b), #4, #5, #6, #7, #8, #9 ^2^
1997.02.26	Lowered the threshold amount of ACMs to 1% *w*/*w* Banned all use of crocidolite and amosite with exemption for #1
1998.07.07	Added one authorized operation: #10 ^2^
1998.12.01	Asbestos should be stored in closed places and containers to prevent scattering
2005.12.30	Announced the prohibition of operation #3(b) and #10 (effective on 2008.01.01)
2009.07.31	Announced the prohibition of operations #2, #4, #5, #6 and #9 (effective on 2010.01.01)
2012.02.02	Announced the prohibition of operation #8 (effective on 2012.08.01) Announced the prohibition of operation #3(a) (effective on 2013.02.01) Announced the prohibition of operation #7 (effective on 2018.07.01)
2013.01.24	Announced the prohibition of operation #3(a) (effective on 2013.02.01)
2017.05.01	Preponed the effectiveness of prohibition of operation #7 (effective on 2018.01.01), but obtained permits will be still effective until expiration of permit

^1^ Authority is Taiwan Environmental Protection Administration. ^2^ Operations are denoted as following purposes: #1 = research, testing, or education; #2 = manufacturing asbestos adhesives or glues; #3(a) = manufacturing asbestos roof tiles; #3(b) manufacturing asbestos plate, pipe, and cement; #4 = manufacturing fireproof, heat resistant, thermal insulation materials containing asbestos; #5 = manufacturing asbestos tape, cloth, rope, or gasket; #6 = manufacturing asbestos filter or asphalt (filler); #7 = manufacturing asbestos containing brake lining; #8 = manufacturing sealant tape for building materials; #9 = manufacturing anti-corrosion paint containing asbestos; #10 = manufacturing fiber cement boards.
